# Atlanta Medical and Surgical Union

**Published:** 1885-02

**Authors:** 


### ATLANTA MEDICAL AND SURGICAL UNION.

Reported for THE ATLANTA MEDICAL AND SURGICAL JOURNAL.

STATED MEETING, December, 1884.

Dr. J.D. Wilson, President, in the chair.

Dr. J. McF. Gaston being present as a visitor, was invited to communicate anything of interest connected with his cases, and responded as follows:

*Mr. President and Gentlemen of the Society:*

Having seen an interesting case of obstetrics during the past week, in consultation with Dr. G. G. Roy, it may prove satisfactory to all present to give its details. Being called on Friday night, or rather after midnight on Saturday morning, the 13th inst., with the intimation to bring chloroform, I learned upon arrival at the bedside of the patient, that my colleague had been in attendance since before day on the previous morning; and that little progress had been made in a breech presentation, with active labor-pains continuing up to that time. Upon a careful examination the diagnosis of breech presentation with the sacrum to the pubic arch of the mother was confirmed, and the buttocks of the child lay in the inferior strait near the vulva, but without protrusion of the perineum, or the least indication of descent on the recurrence of the pains. Being convinced, from retaining the hand in contact with the presenting part for a considerable time, that no progress was made, I acquiesced in the proposition of Dr. Roy to carry out the use of the anæsthetic, which he had already used to a limited extent, for the purpose of exploration; and as a preliminary step the catheter was introduced into the bladder with the discharge of only a small quantity of urine, as it had been passing involuntarily previously, under the rigorous contractions of the womb and the abdominal muscles so as to evacuate the bladder.

While a mixture of chloroform and ether was inhaled freely under the care of Dr. Roy, so as to induce a state of extreme anæsthesia, and thus bring about not only the unconsciousness of the patient, but an interruption of the uterine contraction, with relaxation of the soft parts at the outlet of the vagina, the breech was thrust upward by the hand, which was then passed into the cavity of the womb without much difficulty; but upon reaching the chest of the fœtus, a close constriction of the circular fibres of the uterus was encountered around the body and the extended legs, which lay in contact with the same throughout its front aspect. This cord-like stricture was such that only a single finger could at first penetrate between it and the fœtus, but introducing one finger after another until all entered in a conical shape, the hand was carried around so as to dilate the constricting band until this portion of the inner surface of the uterine wall corresponded to the general relaxed condition of the organ under the influence of the anæsthetic. In then attempting to flex the leg upon the thigh of the child, I found that my fingers were so fatigued by the previous manipulations that it would be impracticable for me to effect it, and yielded my place to Dr. Roy, who succeeded after considerable effort in bringing down the feet. At this point the anæsthetic was suspended, and the uterine contractions having returned, he exerted traction upon the lower extremities, until the descent of the arm-pits to the outlet, where it was arrested, and the arms were found to be doubled up by the sides of the head, thus increasing materially the diameter of the parts engaged in the strait, so that his efforts were directed to bringing down the arms. But, as had occurred with myself previously, he now found his hand so fatigued as to unfit him for this work.

In his turn he now gave way for a resumption on my part of the manipulations, and eventually I succeeded in dislodging both arms, so as to bring them down on tne body, where two fingers were hooked within the lower maxillary of the child, and by steady traction, the body being simply held up out of the way by my colleague, the delivery was effected. It was observed in the descent of the head that the uterine contractions assisted in its expulsion, so that the traction was entirely withdrawn before the final expulsion of the cranial mass.

The child being apparently lifeless, without pulsation in the cord, insufflation of the lungs with a tube was undertaken in conjunction with expulsion of the air by pressure upon the thorax, and subsequently the movements from the prone to the supine position alternately, with a view to induce artificial respiration; but no effect being produced, the cord was divided so as to allow the flow of blood, and the child placed in hot water, friction being resorted to upon the head and chest with brandy.

All was unavailing for its restoration, and having ascertained by the use of the hand in the Crede method that the uterus was well contracted, Dr. Roy proceeded to remove the placenta, leaving our patient free before she returned to consciousness. The usual broad bandage over the abdomen, with a perineal compress secured to the vulva by a strap, completed the immediate requirements in the case, and having aroused from the effects of the anæsthetic the patient was given a cup of tea before we took our leave.

In connection with this rare case of ante partem hour-glass contraction, it may be stated that it is the first instance in which it has been encountered by me in a considerable experience with obstetric cases, and I have only noted in the current literature of the past decade reports of three cases, and neither of them in which the head was retained within the upper segment of the uterus as in this case.

On the other hand, my opportunities for observing the hour-glass contraction after the delivery of the child have been quite extensive, and though Bedford and some of the older writers have expressed doubts as to the existence of post partem hour-glass contraction, there are numerous instances reported by competent observers of this abnormal and irregular spasm of the circular fibres of the womb, securing a greater or less portion of the placenta within the upper division of the cavity of this organ. It may be stated that in all the cases under my observation, which exceed twenty in number, the dilatation has been effected with great effort by the introduction of the fingers gradually, one after another, until the hand assumed a conical form, and when entirely overcome, there seemed to be no disposition to recurrence. The relaxation of the ordinary contraction of the uterine fibres by an anæsthetic, when carried to an extreme point, does not extend to this spasmodic str cture, and it would seem to depend upon an implication of the organic system of nerves, which is not reached by chloroform.

It is claimed by Fancourt Barnes that the nitrate of amyl is available for the relaxation of this spasm of the circular fibres of the womb in hour-glass contraction, but I have had no occasion to verify this observation of its effects.

Dr. Noble wanted to know if there was any rigidity in the perineum of the above case? Dr. Gaston stated that there was none whatever.

Dr. Scott asked Dr. Gaston if he had employed any traction in his endeavors to deliver the child? He said that the constriction was so strong, it was evident that no amount could have accomplished any good.

Other questions were asked by several members present, but no more points of interest were elicited.

Dr. Greer reported a case of labor he had been called to see a few evenings ago. The child was large, but was born without much difficulty. The uterus contracted and the placenta was being expelled when a spasm of the circular fibres of the lower segment of the womb occurred and prevented the complete expulsion of the placenta. At this time the fundus of the womb became relaxed, and considerable occult hemorrhage took place. Chloroform was immediately given to complete narcosis, with the effect of causing a relaxation of this spasm and a normal contraction of the womb without further trouble.

Dr. Gaston wanted to know if the contraction was in the neck or above this portion of the womb, and also asked if he had used counter pressure with the disengaged hand? Dr. Greer stated that the contraction was in the neck, and greatest at os, tightly constricting at this point that portion of the placenta that had passed through the mouth of the womb. In answer to Dr. Wilson's question concerning the condition of the os at the beginning of a-bor, Dr. Greer stated that it was dilated and relaxed, and not rigid.

Dr. R. O. Cotter reported two cases in which he had used the hydrochlorate of cocaine with happy results.

On December 8th Mr. K. aet. 23, called at his office. Right eye was pierced by a knife blade ten years ago. Ball shrunken to two-thirds of its normal size. As the cornea was intact an artificial eye could not be worn. He injected with hypodermic syringe about twenty drops of a 4 per cent. solution of hydrochlorate of cocaine into the tissues around the ball, around the optic nerve and among the ciliary nerves as well as possible. He then made the enucleation in the usual manner. The operation caused very little pain. The patient stated that the pressure of the speculum caused more pain than the operation itself.

Case 2d—Mr. G., aet. 49, blacksmith, called December 13th. Left eye, irido choroiditis and dislocated lens, result of a blow twenty years ago. Sympathetic symptoms began in right eye several months since, and vision is now so seriously impaired as to greatly interfere with his work. He dropped a few drops of a 4 per cent. solution of cocaine in left eye, and after waiting four minutes, injected about half drachm of the same into the tissue close to the ball, carrying the point of the needle as well as possible towards the optic nerve. Owing to the full size of the ball, it was more difficult to carry the solution to the tissue behind the ball than it was in case No. 1. In this case about four insertions of the needle were made; one at about the point of insertion of each of the four recti muscles. The ball was then enucleated in the usual manner. In this case a little more pain was experienced by the patient than in previous case. Yet, after the operation the patient stated, as did the first one, that the speculum caused as much or more pain than the operation. These two cases prove conclusively to my mind the superiority of cocaine in cases of enucleation (in adult patients) over chloroform or ether, and I shall hereafter use the cocaine.

Dr. H. F. Scott thought no more important discovery had been made for years than the uses to which hydrochlorate of cocaine could be put, and that it was destined to revolutionize operations on the eye, ear, throat and nose, and in fact all minor surgical operations in all other departments.

He had made use of a 2½ per cent. solution to facilitate the removal of foreign bodies from the conjunctiva and cornea and tumors from the lids—all done with practically no inconvenience or pain to patient.

In an obscure case of otalgia, with no appreciable cause, he had dropped from 10 to 15 drops of this solution into the auditory canal, and then plugged with cotton; patient was soon relieved and has had no recurrence.

Saw patient the following morning, and on inspection of membrana tympani and lining membrane of canal, found no irritation or congestion of parts; thus farther demonstrating the innocuous and unirritating qualities of a pure article.

In the case of an exceedingly nervous lady, whose fauces were so irritable as to prevent even the slightest examination of the throat —not even tolerating depression of the tongue—he had succeeded in satisfactorily examining the case, by first penciling base of tongue and the anterior fauces, and after a short interval touching the deeper structures with this solution.

In a very severe case of toothache, where the nerve was exposed, plugging the cavity with cotton saturated with the cocaine solution had a magical effect.

Dr. Scott had, also, great faith in the good results likely to follow its use in operations for phimosis. He felt confident that if, in such cases, it were well penciled on integument and then thoroughly injected around the glans penis, so as to be brought directly in contact with the preputial lining membrane, repeating these applications several times at intervals of a few minutes, and maybe, in some cases, injecting a few drops into the redundant tissues, the sensibility could be completely obtunded, thus obviating the pain ordinarily attending this operation, and also the risks and inconveniences of chloroform and ether.

He had tried in only one case of circumcision in a child one and a half years old, and had made the section of the tissues and slit up the mucous membrane, with evidently much less pain than ordinarily accompanies such procedures; but, in this case, on account of the firm adhesions between glans penis and prepuce in almost their entire extent, he was finally compelled to chloroform the patient ere they could be safely separated and the sutures placed.

Dr. Howell reported a case in which he had used the cocaine in cutting a stricture situated in urethra one inch from meatus. He injected five drops of the solution with a p. p. syringe into the urethra and retained it there for four minutes. The injections were repeated three times, and at the expiration of twelve minutes the meatus was enlarged and the stricture divided to full size of urethral calibre. The pain was very slight.

Dr. Elkin reported two cases, in which he had used hydrochlorate of cocaine with marked effect. Nov. 1st, Mr. C. M. called at his office to consult him in regard to a lesion situated just within the meatus urinarius. He examined the part affected and found a large destructive chancroid in the roof of the urethra, just within the meatus—spreading rapidly forward and backward, and threatening perforation of the anterior portion of the glans penis. He decided to cauterize the same; and knowing that considerable pain would be experienced from the nitric acid that he intended to use, he secured a 4 per cent. solution of hydrochlorate of cocaine and applied it in the following manner. The chancroid which extended backward and forward one half an inch within meatus and occupied the roof of the urethra at this point, was readily exposed by an ordinary urethral speculum and the lesion was thoroughly cleansed and dried by means of absorbent cotton With an ordinary dropper six or eight drops were placed upon the exposed chancroid and allowed to remain fully five minutes. At the expiration of that time the solution was wiped off with absorbent cotton, and two other applications made in a similar manner. In fifteen minutes from the time of first application the nitric acid was applied, scarcely any pain being complained of. When the cocaine was first applied the patient experienced a tingling and prickling sensation, similar to that of aconite, and the color of the chancroid assumed a whitish appearance, resembling a mucous membrane that had been subject to an application of carbolic acid. The cauterization of the parts was complete and the same healed readily in the course of two weeks. Solution carbolic acid and iodoform constituted the after-treatment.

Second Case. G. W. G. came to his office on the morning of Nov. 10. On examination there was found to be three chancroids on the mucous-membrane of the prepuce. The edges were ragged and undermined, and the lesions were rapidly spreading.

Dr. E. does not believe in burning every suspicious looking ulcer that presents itself to him for treatment, but he was so well pleased with the effects of the hydrochlorate cocaine in the case that has just been reported that he was persuaded to try it again. The chancroids were cleansed and dried and the surface of each was covered with the solution, and repeated in the same manner as in the previous case. In eighteen minutes from the first application carbo-sulphuric paste was applied. The pain in this case was much lessened but not entirely absent. The patient, however, who had had previous attacks of chancroids cauterized, was much gratified to find the pain so slight and trifling as compared to suffering experienced on former occasions.

The third case reported by Dr. Elkin was the rapid dilatation of a stricture, one and a half inches from external meatus. The patient, Mr. H., had been troubled for the past two years with a chronic discharge from the urethra, and upon careful examination with Otis' urethrometer the presence of a stricture was detected at the points mentioned above. The calibre of the stricture would only admit of the passage of a number 22 French sound, and so irritable and sensitive was the mucous membrane of the canal, that the introduction of the sounds would cause much pain. He was anxious to etherize the patient and divide the stricturous band by means of Otis' urethrotome, but the patient would neither consent to take ether nor submit to the use of the knife. He consented, however, to try the cocaine and have the stricture dilated by means of Thompson's dilator. He secured a deep urethral syringe and injected twenty drops of the 4 per cent. solution of the hydrochlorate of cocaine into the urethra at the seat of stricture, having the patient to close the urethra just below the stricture with the thumb and fore finger of one hand. After injecting the solution Dr. E. pressed the lips of the external meatus tightly together so as to prevent the escape of the fluid on removal of the syringe. The injections were repeated twice and retained in the urethra for ten minutes each time, and after this the dilator was immediately introduced and the stricture slowly and gently divulsed to the normal calibre of the urethra. The pain accompanying the operation and the subsequent introduction of the sounds up to 32 mm., was not at all severe. Dr. Elkin thinks the drug will prove an invaluable aid in genito-urinary surgery. He believes it will completely mask the pain attendant upon the use of caustics and greatly lessen the pain experienced from the introduction of sounds into an irritable and sensitive urethra.

